# Work ability and associated factors among individuals with Post COVID-19 condition- a cross-sectional study

**DOI:** 10.1186/s12889-026-26771-0

**Published:** 2026-02-28

**Authors:** Kjerstin Stigmar, Iben Axén, Christina Brogårdh, Elisabeth Ekstrand, Agneta Malmgren Fänge, Eva Ekvall Hansson

**Affiliations:** 1 Department of Health Sciences, Lund University, Lund, Sweden; 2 Intervention and Implementation Research for Worker Health, Institute of Environmental Medicine, Karolinska Institutet, Stockholm, Sweden; 3 Department of Neurology, Rehabilitation Medicine, Memory Disorders and Geriatrics, Skåne University Hospital, Lund, Sweden; 4 Department of Hand Surgery, Skåne University Hospital, Malmö, Sweden; 5 Ear-Nose- and Throat Department, Skåne University Hospital, Lund, Sweden

**Keywords:** Work ability, Post COVID-19 condition, Mental fatigue, Physical fatigue

## Abstract

**Purpose:**

Long-standing symptoms have been reported after COVID-19 infection, which can negatively impact daily life and work. The purpose of this study was therefore to describe work ability and explore factors associated with poor work ability among individuals with Post COVID-19 Condition (PCC).

**Methods:**

We did an online survey among individuals with self-reported PCC. Descriptive statistics and multivariate logistic regression analysis were used to explore relationships between different factors associated with poor work ability.

**Results:**

A total of 608 individuals answered the survey (mean age 47.3 years (SD 9.9), 87% women). The majority (85%) had not been hospitalized on account of a COVID-19 infection. Work ability was experienced as poor among 51%, but only 18% were on sick leave. In the final logistic regression model of four variables (Nagelkerke R Square 0.538, *p* < 0.001), mental fatigue was the most important factor for perceived poor work ability (Odds ratio 1.176; CI 95%: 1.123–1.232 *p* < 0.001, Nagelkerke R Square 0.406). Higher levels of dependency in Activities of Daily Living (ADL), greater physical fatigue, and impaired balance were also associated with self-reported poor work ability.

**Conclusion:**

Perceived poor work ability is common among people with PCC. Several factors are associated with poor work ability in this population whereof mental fatigue seems to be strongest. Given the complexity of these conditions and the consequences in everyday life, it is crucial to implement individually tailored interventions.

## Background

The COVID-19 pandemic had by December 2025 affected nearly 800 000 000 individuals and caused more than 7 million deaths globally [1]. Most of the infected individuals were not severely ill or in need of hospital care; instead, a non-negligible proportion experienced long standing symptoms after the acute infection [2]. WHO has named this Post COVID-19 condition (PCC) and defined it as “the continuation or development of new symptoms three months after the initial SARS-CoV-2 infection, with these symptoms such lasting for at least two months with no other explanation” [3]. The etiology for PCC is not yet fully understood [4] and heterogenicity in explanations exists. One challenge for better understanding of PCC is that both non-hospitalized and hospitalized individuals are often mixed in studies, and PCC is not always defined in the same way from study to study [5]. WHO has reported that around 10–20% of patients with COVID-19 develop PCC, but it seems like this percentage is decreasing [3]. SARS-CoV-2 virus is still active and every new infection with the virus constitutes a risk of developing PCC.

In a systematic review and meta-analysis, Chen et al. [6] studied PCC symptoms among hospitalized and non-hospitalized individuals and found that as many as 43% reported to have PCC and this was more common among hospitalized individuals. In a study among health care workers, the authors found a prevalence of 2.4%, where women of working age were more affected [7]. A diversity of symptoms, such as mental and physical fatigue, dizziness, cardio-respiratory symptoms, and musculoskeletal symptoms are described [5, 8, 9]. Fatigue has been reported to be the most common symptom among individuals with PCC [8, 10–13]. Decreased physical activity and more dependency in activities of daily life (ADL) have also been reported [11, 12, 14]. These different symptoms can have a severe impact on the individual’s activity and participation in daily life, including work. In a longitudinal study that included individuals with PCC from 56 different countries, 70% had not fully returned to work (RTW) [10], which indicates a decreased work ability.

Work is often described as a component of health [15, 16] and accordingly it is important that individuals of working age can partake in working life. A recent study found that decreased self-reported work ability, defined as present work ability compared to life-time best, was the factor strongest associated with experiencing low life satisfaction after COVID-19 [8]. In addition, Lemhöfer et al. [17] found that perceived work ability had an impact on health-related quality of life among patients with PCC. A recent systematic review concluded that a considerable proportion of individuals (29–47%) were not able to return to work (RTW) after a COVID-19 infection [5]. Kerksieck et al. reported that 5.8% of a cohort of individuals with PCC reported PCC symptoms at work and it was also more common to report poor work ability among individuals with PCC [18]. In a Swedish register-based study of individuals with PCC, 13.3% were on sick leave [19] and age, recent sick leave and hospitalization increased the risk for sick leave. Among those who were on sick leave during the first wave of COVID-19 pandemic in Sweden, 85,6% experienced functional limitations at 18 months follow-up [13]. These individuals are thus excluded from work, which may have a substantial negative impact on their daily life, economy and, subsequently, health.

Work ability is complex concept and encompasses individual aspects such as physical, psychological, cognitive, social/behavioral factors but also workplace factors, and factors outside the workplace [20]. Factors such as age, health status and type of work organization have been found associated with self-reported work ability [21] and this was very much in line with the results from a systematic review from van den Berg et al. [22]. Reduced work ability may result in sick leave, and this is a common problem among individuals with PCC and in a Danish cross-sectional study, 56% reported to be on full-time or part-time sick leave due to PCC [23]. Self-reported work ability reflects the individual´s experience of their ability to work, not necessarily work absence. Thereby an individual with decreased work ability can still be at work, while limitations in work performance are present, i.e. presenteeism [24].

Interventions to improve work ability are often directed towards the individual and include different types of health focused interventions. However, a systematic review [25] emphasizes the importance of multidomain interventions, also targeting the workplace, for individuals with musculoskeletal pain or mental health problems in order to enable a successful RTW. In a cohort study, Brehon et al. [26] emphasized the importance of being able to modify work tasks for individuals with PCC, to support RTW. Sulg et al. [27] interviewed 32 individuals with PCC about RTW after PCC, and the importance of work flexibility was emphasized.

Possibilities to modify work have been described as adjustment latitude [28]. Adjustment latitude covers if and to what extent there are possibilities to make workplace adjustments that fit an individual´s needs, and furthermore, what type of adjustments are possible. Examples of such adjustments can be to postpone work, work at a slower pace or reduce working hours. In general, individuals experiencing low adjustment latitude at work have been found to be on sick leave to a high extent [29, 30], and employees on long-term sick leave reporting low adjustment latitude were less likely to RTW, both full time and part-time [31]. Generally, adjustment latitude varies between professions and is limited to many types of professions [32]. Since flexibility at work is important to support RTW for individuals with PCC [27], adjustment latitude is a concept that is important to apply.

Work ability has been found to have a strong impact on life satisfaction among patients with PCC [8]. Individuals with PCC have reported lower work ability and taking more sick leave [7, 33]. It is important to study both individual factors and factors at work, that can be associated with poor work ability. Today, the incidence of PCC after an acute COVID-19 infection appears lower, but still, the virus is present and thereby there is a risk of developing PCC [3]. Therefore, the aim of this study was to describe work ability and explore factors associated with poor work ability among individuals with Post COVID-19 Condition (PCC).

## Methods

### Study design and context

This is an explorative cross-sectional study, using survey data from a larger project “Life After COVID-19 (LAC) in Sweden”. The LAC project aimed at exploring the perceived consequences of symptoms after COVID-19 [8, 34–36]. After completing a baseline survey, the participants in the LAC-project were invited to take part in a longitudinal study by answering short-text messages for 52 weeks (*n* = 344). In this present study we use baseline survey data only.

### Recruitment and participants

Participants were recruited through social media in October and November of 2021. Inclusion criteria were adults (minimum 18 years), able to read and write Swedish, with self-reported symptoms after COVID-19 infection lasting more than two months. Thus, participants were not included based on a specific set of symptoms. In total, 766 individuals answered the survey. The recruitment procedures are explained in detail elsewhere [8].

### Data collection

After giving informed consent, eligible participants answered a digital questionnaire hosted on the website of Lund University. The questionnaire was constructed for the LAC project and consisted, in general, of validated scales and questionnaires as described below. The construction was guided by Wade [37] to cover relevant symptoms and outcome measures. The baseline questionnaire comprised several items relating to the initial COVID-19 infection (onset, symptoms and need for hospital care), post-COVID-19 symptoms (perceived physical and mental fatigue, dizziness and balance, activities of daily living (ADL), aerobic capacity, work ability, and life satisfaction), and the perceived consequences thereof. Sociodemographic variables were collected (age, sex, family situation, residence, type of profession, education) as well as the presence of comorbidities.

In our dataset, health was described by the duration of PCC-symptoms (months) and the presence of comorbidities (yes/no). Perceived functioning and disability were covered by health-related questionnaires. Mental fatigue was reported with Mental Fatigue Scale [38], where a total score ≥ 10.5 indicates mental fatigue. Physical fatigue was reported by the Fatigue Severity Scale (FSS) [39], where a score ≥ 4 signifies physical fatigue. Experience of dizziness and balance disturbance was reported with a dichotomous question, respectively (yes/no). Level of dependence in ADL was reported using the total score of the ADL Staircase [40, 41], where a higher value indicates higher dependence (0–30). For aerobic capacity, we used a rating scale [42]. A high value (20 for men, 18 for women) indicates high aerobic capacity. The questionnaires are described in detail previously [8]. To describe possibilities to modify work, two questions regarding adjustment latitude at work were included; how often adjustments were possible (never/seldom/sometimes/often) and the number and type of adjustment options (0–9).

The Work Ability Index single item question (WAS) was used to measure the self-perceived ability to work [21]. The WAS measures the perceived current work ability in relation to the individual’s lifetime best and ranges from 0 to 10. The score was further categorized as poor (0–5 points), moderate (6–7 points), good (8–9 points) or excellent (10 points). This instrument has been found valid and reliable (ICC 0.89) for assessing work ability in research [21, 43, 44].

### Data analysis

SPSS version 28.0 was used for the analysis (IBM Corporation, Armonk, New York, NY, USA). Background characteristics and self-reported outcomes were presented with mean and standard deviation (SD) or with median and interquartile range (IQR), furthermore categorized or dichotomized in number and frequencies, using descriptive statistics.

Work ability was dichotomized into poor and moderate/good/excellent, and logistic regression analyses were used to explore factors associated with poor work ability. The selection of the explanatory variables was guided by expert knowledge and by variables reported in previous literature [37]. The following explanatory variables were used in the present study: age, education (primary, secondary and higher education), duration of PCC-symptoms, comorbidities (yes/no), fatigue severity score (FSS score), mental fatigue score (MFS score), dizziness (yes/no), impaired balance (yes/no), aerobic capacity (Borg´s RPE score), ADL dependence score (ADL Staircase score), and adjustment latitude (never vs. seldom/sometimes/often) and number of adjustment options.

The multivariate regression modelling was performed using a generous inclusion criterion (*p* ≤ 0.20) to ensure that potentially relevant variables were not excluded at an early stage. Univariable analyses were first conducted to evaluate associations with workability. Odds ratios (OR) with 95% confidence intervals (CI), explanatory value (Nagelkerke R²), and p-values were calculated for each variable. The variable with the lowest p-value value was included as the initial variable in the model. Stepwise model building then proceeded by tentatively adding variables with *p* ≤ 0.20 in the univariable analyses, one at a time. After all candidate variables had been tested, the two-variable model with the highest Nagelkerke R² was retained. The process was iteratively repeated, adding remaining variables one at a time and retaining the combination that maximized explanatory value, as long as the model remained statistically significant and that the Nagelkerke R² increased. In the final model, only variables with *p* < 0.05 were retained. This selection strategy in the model building was chosen to enhance understanding of the relative significance of the different variables, allowing the identification of those with the greatest explanatory contribution while maintaining a transparent and interpretable model.

## Results

In total, 608 individuals had answered all explanatory factors and were included in the study (Table 1). Their mean age was 47 years (SD 9.9), and 87% were women. Of the participants, 73% had a higher education and 70% were working, while 18% reported to be currently on sick leave. The majority (85%) did not need hospital care due to the COVID-19 infection.

**Table 1:** Characteristics of the study sample (*n*=608)

	n (%)	Mean (SD)
Age		47.3 (9.9)
Sex		
Men	72 (12)	
Women	528 (87)	
Other/do not want to report	8 (1)	
Educational level		
Primary (8-9 years)	7 (1.0)	
Secondary (10-12 years)	159 (26)	
Higher education (college/university)	442 (73)	
Provision		
Work	428 (70.4)	
Student grants	15 (2.5)	
Sick leave benefit	112 (18.4)	
Unemployment benefit	9 (1.5)	
Social security benefit	2 (0.3)	
Other sources of income	42 (7)	
Duration of post COVID-19		12.8 (5.0)
Comorbidity Yes	236 (39)	
Hospitalization for COVID-19		
Intensive care	11 (1.8)	
Other department	76 (12.5)	
No	517 (85)	
Missing	4 (0.7)	

Self-reported outcomes are described in Table 2. Work ability was experienced as poor among 51% of the participants. Regarding fatigue, 83% experienced mental fatigue and 86% physical fatigue. Dizziness was reported by 84% of the individuals and balance disturbance among 55%. ADL dependence was reported as low (median 2, IQR 0–5) as was also physical capacity (median 5, IQR 4–7).

**Table 2:** Self-reported outcomes at baseline *n*=608

	n (%)	Median (IQR)
Mental fatigue^a^		18 (12.5-22.5)
Mental fatigue score ≥10.5	507 (83)	
Physical fatigue^b^		6.1 (4.9-6.7)
Physical fatigue score ≥4	525 (86)	
Dizziness- yes	504 (83)	
Balance- impaired -yes	333 (55)	
Activities in Daily Living^c^		2 (0-5)
Physical capacity^d^		5 (4-7)
Work ability^e^		5 (3-7)
Work ability		
Poor	310 (51)	
Moderate	163 (27)	
Good	112 (18)	
Excellent	23 (4)	
Adjustment latitude		
Never	171 (28)	
Seldom	103 (17)	
Sometimes	177 (29)	
Often	157 (26)	
Adjustment alternatives*		
Postpone work	229 (38)	
Choose between different work tasks	140 (23)	
Get help from colleagues	149 (25)	
Work at slower pace	158 (26)	
Take longer breaks	138 (23)	
Work fewer hours	140 (23)	
Go home and do some work later	86 (14)	
Work uninterupted	51 (8)	
Work from home	221 (36)	
Number of adjustment options		
No options	196 (32)	
1	190 (31)	
2	56 (9)	
3	57 (9)	
4	31 (5)	
5	21 (3)	
6	27 (4)	
7	13 (2)	
8	17 (3)	
9	0 (0)	

* More than one option possible

^a^Mental Fatigue Scale; ^b^Fatigue Severity Scale; ^c^ADL Staircase; ^d^Borg´s RPE Scale; ^e^Work ability Score

More than half of the participants reported that workplace adjustments were possible sometimes (29%) or often (26%), but for 17% it was only possible seldom and for the remaining 28% never. The majority (63%) reported no or only one adjustment option, and the most common adjustment alternatives were to work from home, postpone work or to work at a slower pace (Table 2).

In the univariate regression analyses, all variables were associated with poor work ability (*p*-value ≤ 0.20), except age (Table 3), and therefore included in the multivariate model building. In the final multivariate regression model (Table 4), mental fatigue had the highest value of explanation (OR 1.176; CI 95%:1.123–1.232; Nagelkerke R Square: 0.406). The final model also included ADL dependence (OR 1.344; CI 95%:1.244–1.452), physical fatigue (OR 1.378; CI 95%:1.115–1.702) and experiencing impaired balance (OR 1.920; CI 95%: 1.202–3.068). ADL dependence added 0.11 and physical fatigue and impaired balance 0.01 each to the total explanatory value (Nagelkerke R Square 0.538, *p-*value < 0.001).

**Table 3:** Univariate logistic regression analyses of factors associated with poor work ability (*n*=608)

Explanatory factors	*p-value*		Odds ratio (95% CI)	Nagelkerke R Square
Age	0.292		1.009 (0.993-1.025)	0.002
Educational level (ref higher vs lower)	0.128		1.321 (0.923-1.891)	0.005
Comorbidities (ref no vs yes)	0.003		1.637 (1.178-2.276)	0.019
Duration of PCC symptoms (time)	0.018		1.039 (1.007-1.073)	0.012
Physical fatigue (FSS score)	<0.001		2.384 (1.992-2.854)	0.269
Mental fatigue (MFS score)	<0.001		1.245 (1.200-1.292)	0.406
Dizziness (ref no vs yes)	<0.001		4.373 (2.682-7.131)	0.087
Balance (ref not impaired vs impaired)	<0.001		2.194 (1.584-3.039)	0.049
Aerobic capacity (Borg´s RPE scale)	<0.001		0.691 (0.636-0.751)	0.223
ADL dependence (ADL Staircase score)	<0.001		1.499 (1.389-1.618)	0.374
Adjustment latitude (ref never vs seldom/sometimes/often)	0.077		1.379 (0.966-1.968)	0.007
Number of adjustment options	0.045		0.925 (0.857-0.998)	0.009

**Table 4:** Final multivariate logistic regression analyses of factors associated with poor work ability (*n*=608)

				Cumulative
Explanatory factors		*p-value*	Odds ratio (95% CI)	Nagelkerke R Square
Mental fatigue (MFS score)		<0.001	1.176 (1.123-1.232)	0.406
ADL dependence (ADL Staircase score)		<0.001	1.344 (1.244-1.452)	0.517
Physical fatigue (FFS score)		0.003	1.378 (1.115-1.702)	0.528
Balance (ref not impaired vs impaired)		0.006	1.920 (1.202-3.068)	0.538

*CI* Confidence interval

Nagelkerke R Square value = pseudo R-square value that tells how well the model explains the dependent variable (0-1)

## Discussion

In this study we found that more than half of the participants experienced poor work ability. Despite this, the majority (70%) were working. Mental fatigue was the strongest factor that was associated with poor work ability.

The most prevalent symptoms in our cohort were mental and physical fatigue together with dizziness. Previous studies have shown that fatigue is one of the most common symptoms in people with PCC, both among those who had been non-hospitalized and hospitalized [5, 8, 9, 13], which may impact physical and cognitive functions [8]. In the present study, mental fatigue was the strongest associated factor with poor work ability. This finding is in line with the study by Muller et al. [45], who reported that fatigue is strongly associated with a low work ability and delayed RTW. A recent meta-analysis by Hu et al. [46] found that being female, older age, clinical severity and number of acute COVID symptoms, preexisting, lung disease and depression are risk factors for post-COVID-19 fatigue. There is limited evidence of how fatigue should be treated in people with PCC. However, mindful exercises [47] and pulmonary rehabilitation [48] seem to be promising interventions. As fatigue also is prevalent in the general population, especially among women [49], special attention may be needed towards gender distribution among patients with PCC [13].

For the dependent variable work ability, we used WAS [21]. This reflects the individual´s own perception of work ability and says nothing about work absence. 51% reported poor work ability. This is previously reported to be common among individuals with PCC [18]. In this present study, only 18% reported to be on sick leave, so there are reasons to believe that there is decreased work productivity and presenteeism among the participants. Westerlind et al. [19] studied a large sample using register data and found that 13.3% were on sick leave due to PCC. Unfortunately, we had no information regarding self-reported work productivity or productivity loss reported by employers, or access to register data on sick leave. In a systematic review from 2023, Gualano et al. [50] studied how PCC symptoms impacted RTW after hospitalization due to COVID-19. They highlighted PCC as an important area to address in occupational medicine, since PCC severely impacted work productivity as well as quality of life. Seljelid et al. found that being hospitalized was associated with reporting more problems [13]. Also, Ekstrand et al. [8] found that poor self-reported work ability was that factor which was strongest associated with experiencing low life satisfaction.

Work ability is a complex concept that includes both individual and workplace factors [20]. Therefore, to be able to tailor interventions to retain or maintain work ability it is important to uncover both these aspects. Multidomain interventions, including the workplace, are suggested for individuals with musculoskeletal pain or mental disorders [25]. These types of symptoms are commonly reported in PCC [5, 8, 9]. Possibilities to modify at work [26, 51] and flexibility at work [27] and a supportive workplace culture [51] have been pointed out as important factors to support RTW among individuals with PCC. In this present study the majority (70%) were working and 54% reported access to such possibilities sometimes or often. Low adjustment latitude was not associated with poor work ability. This may indicate that possibilities to adjust at work enabled the study participants to stay at work, even though they were experiencing poor work ability. Standal et al. [52] found that individuals on part-time sick leave (all causes) had better opportunities to adjust work. In our study we have no information regarding full-time or part-time sick leave, but only a minority (18%) were on sick leave.

We also found that ADL dependence, such as cleaning, shopping etc., was associated with poor work ability. These types of activities can be common work tasks in blue collar occupations; however, in the present study most of the participants were highly educated, and we have reasons to believe that they have more white-collar type of occupations. Despite this, it is reasonable to expect that individuals who are dependent on physically and cognitively demanding activities, such as shopping and transportation (travelling to and from work by public transportation or car), may also experience reduced work ability. Similar findings have been demonstrated in previous research targeting individuals with post-COVID conditions [10, 13]. Previous research has indicated limited evidence for the relationship between fatigue and ADL decline among people with stroke [53]. Even if we assume that ADL dependence was not significantly impacting work, experiencing such dependence in private life can be stressful and may impact on how work ability is experienced. The cumulative characteristics of the ADL Staircase used to measure ADL dependence supports this line of reasoning [40, 41].

Impaired balance was also retained in our final model. Individuals that reported greater balance impairments generally had higher odds of perceiving poor work ability. However, its explanatory contribution was small. Balance disturbance can be due to different causes such as dizziness, which has been reported as one of the most common symptoms in PCC [5, 8, 9]. Since dizziness is a rather disabling condition, it would be reasonable to assume that this can impact work ability in a negative way, but in this study, dizziness was not included in the multivariate analyses, even though being significantly associated with poor work ability in the univariate model. There might be other possible explanations for this association, but we had no access to such factors.

### Strengths and weaknesses

Given the necessity of investigating the long-term consequences of COVID-19, we chose to use an exploratory cross-sectional design. Thus, we were able to both describe the outcomes and identify factors associated with poor work ability. We had a large number of participants, used valid questionnaires, and had a low rate of internal missing data. However, a longitudinal design would have enabled us to identify factors predicting poor work ability.

All of our data was self-reported. Participants were included based on self-reported symptoms, which are prone to bias related to recall and social desirability, and reported data may be inaccurate, possibly leading to over- or under-reporting. Work ability was also self-reported and access to register data on sick leave could have increased the trustworthiness of data. Furthermore, we had no information on what type of occupations the participants had. This is of course a shortcoming, and such information could have helped to interpret the results. We know that 73% of the participants had a college/university degree and therefore there are reasons to believe that the majority have cognitively demanding occupations.

Since more women than men were included and the participants in general were highly educated, there are reasons to question the representativeness of our results. Recruiting via social media was an effective strategy to obtain a high number of participants, but it is well-known that the risk for selection bias increases [54, 55]. Women are more frequently on Facebook, but as also found in this present study, PCC is also more common among women ([7, 13]. The small proportion of participants on sick leave also made us unable to perform robust and reliable stratified analyses, comparing those working and those on sick leave.

Another limitation of the study was that the inclusion criteria were not fully compatible with the WHO definition of PCC [3]. According to the WHO, the symptoms should have been present or had arisen three months after the initial SARS-CoV-2 infection and lasting for at least two months, but in this study, we included individuals with symptoms lasting for more than two months after the COVID-19 infection. Data collection was conducted in 2021 and the WHO definition was introduced in December 2022, so it was not possible to apply to the inclusion criteria. A further limitation is that we included both hospitalized and non-hospitalized participants, which seems to be a well-known challenge [5]. In this study, a majority (85%) were not hospitalized. This indicates that the PCC symptoms did not appear after a severe COVID-19 infection that needed specialized care, rather after an infection that was under self-treatment or treated in primary care.

Finally, a limitation of using stepwise regression is that it may be affected by multicollinearity and overfitting, potentially producing sample-specific results. However, we attempted to minimize these effects by guiding variable selection based on prior knowledge and literature and applying a systematic, stepwise approach with generous inclusion criteria, which also allowed the identification of key variables in a transparent and reproducible manner.

## Conclusion

Perceived poor work ability is common among people with PCC. Several factors are associated with poor work ability in this population, whereof mental fatigue seems to be strongest. Given the complexity of these conditions and the consequences in everyday life, it is crucial to implement individually tailored interventions to support individuals towards increased activity and participation in all aspects of life, including work.

## Data Availability

The data used in this study contains sensitive information about the study participants and they did not provide consent for public data sharing. The ethical approval from the Swedish Ethical Review Authority (Dnr 202-02776) does not include data sharing. A minimal data set could possibly be shared on reasonable request sent to the corresponding author, from a qualified academic investigator for the sole purpose or replication of the present study, provided the data transfer agrees with EU legislation on the general data protection regulation (GDPR) and approval by the Swedish Ethical Revies Authority.

## Abbreviations

ADL: Activities of Daily Living
CI: Confidence interval
COVID-19: Severe acute respiratory syndrome coronavirus 2 (SARS-CoV-2)
FSS: Fatigue Severity Scale
ICC: Intraclass correlation coefficient
IQR: Interquartile range
LAC: Life After COVID-19
MFS: Mental Fatigue score
OR: Odds ratio
PCC: Post COVID-19 Condition
RPE: Rating of Perceived Exertion
RTW: Return to work
SD: Standard deviation
SPSS: Statistical Package for Social Sciences
WAS: Work Ability Index single item question
WHO: World Health Organization
